# miR-155 Promotes ox-LDL-Induced Autophagy in Human Umbilical Vein Endothelial Cells

**DOI:** 10.1155/2017/9174801

**Published:** 2017-06-04

**Authors:** Zhaozhi Zhang, Xudong Pan, Shaonan Yang, Aijun Ma, Kun Wang, Yuan Wang, Ting Li, Shihai Liu

**Affiliations:** ^1^Department of Neurology, The Affiliated Hospital of the Qingdao University, Qingdao, Shandong 266100, China; ^2^Central Laboratory, The Affiliated Hospital of the Qingdao University, Qingdao, Shandong 266100, China

## Abstract

As an evolutionarily conserved metabolic process, autophagy is involved in the process of atherosclerosis (AS). MicroRNA-155 (miR-155), a multifunctional miRNA, plays an important role in many physiological and pathological conditions, including AS and autophagy. However, the effect of miR-155 on the regulation of autophagy in endothelial cells has not been reported to date. Therefore, the objective of our study was to investigate the role of miR-155 in autophagy induced by oxidized low-density lipoprotein (ox-LDL) in human umbilical vein endothelial cells (HUVECs). Our results demonstrated that ox-LDL induced autophagy in HUVECs and increased the expression of miR-155 significantly. Overexpression of miR-155 improved autophagic activity, whereas low expression of miR-155 inhibited autophagic activity. Therefore, the data demonstrated that miR-155 has a modulating effect on the autophagy of vascular endothelial cells.

## 1. Introduction

Autophagy is an evolutionarily conserved metabolic process in which denatured proteins and damaged organelles are degraded through the lysosomal system for the maintenance of intracellular homeostasis during various stress conditions [1, 2]. The process is essential for cell growth, differentiation, and development [3]. Accumulating data have indicated that autophagy plays a potential role as diagnostic and prognostic indicators in various diseases containing AS [4–6], which is currently considered a chronic inflammatory disease of the arterial wall.

MicroRNAs (miRNAs) are a class of endogenous noncoding small RNA molecules that regulate gene expression and mediate posttranscriptional gene silencing by combining with the 3′-untranslaed region (3′-UTR) of the target mRNAs [7]. These RNAs are involved in the regulation of multiple cellular processes, including proliferation, differentiation, development, and apoptosis, [8] and have also been associated with AS [9–11] and autophagy [12, 13].

miR-155, a multifunctional miRNA, plays an important role in many physiological and pathological conditions, including AS [14, 15]. Recently, some studies have demonstrated that miR-155 could regulate the autophagy of tumor cells [16] and macrophages [17]. However, the correlation between miR-155 and autophagic activity in endothelial cells has not been reported. Therefore, the goal of this study was to investigate the role of miR-155 in autophagy mediated by ox-LDL in HUVECs.

## 2. Materials and Methods

### 2.1. Cell Culture

HUVECs were purchased from Genechem Co. Ltd. (Shanghai, China). Cells were cultured in Dulbecco's modified Eagle's medium (DMEM) (Hyclone, USA) supplemented with 10% fetal bovine serum (FBS) (Tianhang Biotechnology Co. Ltd., Zhejiang, China) and 100 U/ml penicillin-100 *μ*g/ml streptomycin (Solarbio Science & Technology Co. Ltd., Beijing, China) at 37°C in a humidified 5% CO_2_ incubator.

### 2.2. Cell Treatment

To observe the effect of ox-LDL on autophagy induction and select the optimal time point, we used 100 *μ*g/ml ox-LDL (Yi Yuan Biotech, Guangzhou, China) to stimulate the HUVECs for 6 and 12 h. Control group cells were cultured in complete medium. To demonstrate the role of miR-155 in autophagy, we divided the cells into five groups: the control, ox-LDL, ox-LDL+miR-155 mimic, ox-LDL+miR-155 inhibitor, and ox-LDL+miR-155 negative control (NC). Cells were transfected with miR-155 mimics, miR-155 inhibitors, and miR-155 NC using Lipofectamine 2000 (Invitrogen, USA) according to the manufacturer's protocol. Twenty-four hours after transfection, cells were exposed to ox-LDL (100 *μ*g/ml). The exposure time was the optimal time point selected by the experiment. Control group cells were cultured in complete medium.

### 2.3. Transmission Electron Microscope

Cells harvested by trypsinization were fixed with 3% glutaraldehyde and postfixed in 1% osmium tetroxide. After dehydration in a graded series of ethanol, cells were embedded in Araldite. Next, 50 nm ultrathin sections were placed on nickel grids and subsequently stained with uranyl acetate and lead citrate.

### 2.4. Quantitative Reverse Transcription Polymerase Chain Reaction (qRT-PCR)

Total RNA was extracted from cells using Trizol reagent (Invitrogen) according to the manufacturer's protocol. qRT-PCR analysis for miRNAs was performed by using a miRNA cDNA Synthesis Kit and miRNA qPCR Assay Kit (ComWin Biotech Co. Ltd., Beijing, China). qRT-PCR analysis for mRNAs was conducted according to manufacturer's instructions using a PrimeScript™RT reagent Kit and SYBR®Premix Ex Taq™ (Takara, Japan). All primers used in the study were obtained from Sangon Biotech, Shanghai. Relative expression levels were calculated using the 2^−△△Ct^ method. The mRNA level of GAPDH was used as an internal control.

### 2.5. Western Blot

Cells were washed twice with PBS and then lysed in RIPA buffer (Takara, Japan) containing the protease inhibitors. After centrifugation at 12000*g* for 20 minutes at 4°C, total protein concentration in the supernatant was quantified using the BCA protein assay kit (Comwin Biotech Co. Ltd., Beijing, China). Protein samples were separated by SDS-PAGE electrophoresis and then transferred onto PVDF membranes (Millipore, USA). The membranes were blocked with 5% nonfat milk powder-TBS-Tween 20 for 2 hours at room temperature and then incubated with 1 : 1000-diluted primary antibodies against LC3 and p62 (Cell Signaling Technology, USA) overnight at 4°C. Then, membranes were briefly washed thrice with TBST and incubated with 1 : 10000-diluted horseradish peroxidase-conjugated secondary antibody (Abcam, USA) for an additional one hour at room temperature. After washing thrice with TBST, the membranes were visualized using an ECL Western Blotting Substrate Kit (Millipore, USA) and the GelDoc XR Gel Documentation System (BioRad, USA). The gray value was analyzed by ImageJ software.

### 2.6. Statistical Analyses

Data were presented as the mean ± SD for at least three sets of separate experiments. Differences between groups were assessed using one-way ANOVA with a Tukey correction. Statistical analysis was performed using SPSS 19.0 for Windows software (SPSS Inc., Chicago, IL). *p* < 0.05 was considered to be statistically significant.

## 3. Results

### 3.1. Autophagy Induced by ox-LDL in HUVECs

**Figure 1:**
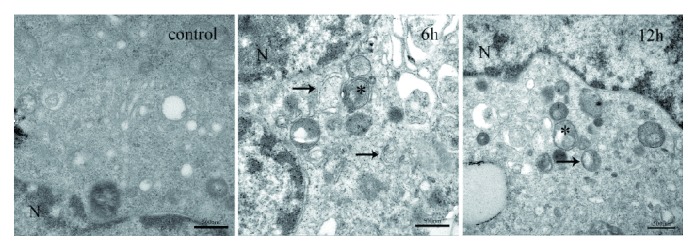
TEM images of HUVECs. HUVECs were exposed to ox-LDL (100 *μ*g/ml) for 6 h and 12 h, and cell samples were collected for TEM analysis. Autophagosomes were indicated by arrows. Autolysosomes were indicated by asterisks. N: nucleus. Scale bar = 500 nm.

**Figure 2:**
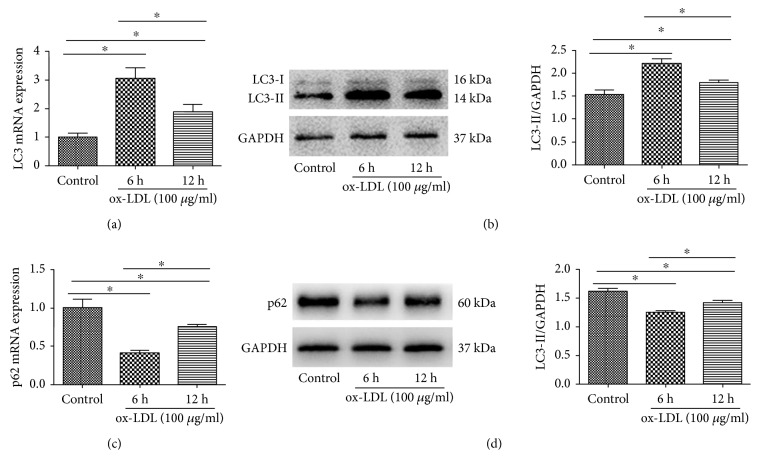
ox-LDL regulates LC3 and p62 expression. HUVECs were treated with ox-LDL (100 *μ*g/ml), and cells are collected at 6 h and 12 h for qRT-PCR and Western blot. (a, c) qRT-PCR analysis of LC3 and p62. Results are the means ± SD of three separate experiments,^∗^*p* < 0.05. (b, d) Western blot analysis of LC3-II and p62. Left panels present representative blots of LC3-II and p62; right bar graphs showed statistics of optical density measurements. Results are the means ± SD of three separate experiments,^∗^*p* < 0.05.

To observe the autophagic activity, we used transmission electron microscopy (TEM) to detect the autophagosomes and autolysosomes in cells. The autophagosome, a special vesicle with a double membrane structure, is an important marker of autophagy activation, which ultimately fuse with the lysosome to form the autolysosome, leading to the degradation of cellular structures [18]. The control group image displayed few autophagosomes and autolysosomes. In contrast, cells treated with ox-LDL for 6 and 12 h exhibited many typical autophagosomes and autolysosomes in the cytoplasm, and the number of autophagosomes at 6 h was much more than that at 12 h (Figure 1). LC3-II is a well-known marker of autophagy. p62, a polyubiquitin-binding protein, is selectively incorporated into autophagosomes through direct binding to LC3 and efficiently degraded during autophagy [19]. To further confirm autophagy, we observed whether LC3-II and p62 were regulated in response to stimulation by ox-LDL. The results showed that ox-LDL increased the expression of LC3-II but decreased the expression of p62, as shown in Figure 2. And the level of LC3-II at 6 h was much higher than that at 12 h (*p* < 0.05), whereas the level of p62 at 6 h was significantly lower (*p* < 0.05). Therefore, we choose 6 h as the optimal time point for the subsequent experiments.

### 3.2. ox-LDL induced miR-155 Upregulation

**Figure 3:**
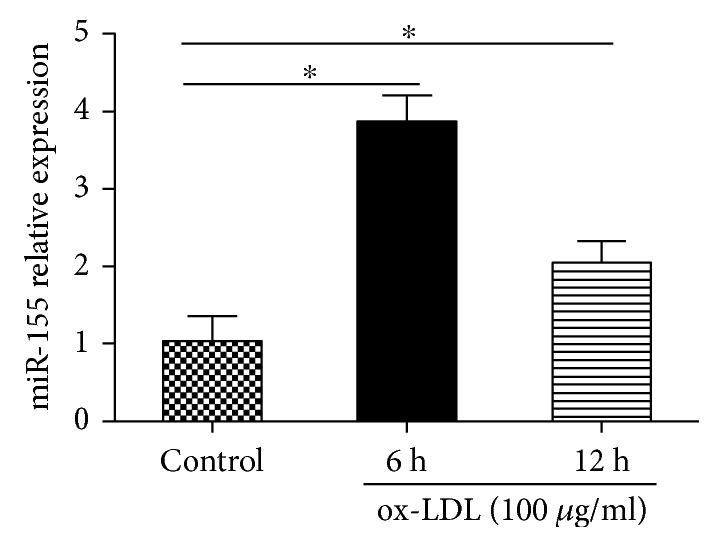
ox-LDL upregulated miR-155. HUVECs were exposed to ox-LDL (100 *μ*g/ml) for 6 and 12 h. Cells were harvested for qRT-PCR to quantify miR-155 expression. Results are the means ± SD of three separate experiments, ^∗^*p* < 0.05.

To explore the relationship between ox-LDL and miR-155, we measured miR-155 expression levels in cells treated with ox-LDL (100 *μ*g/ml) for 6 and 12 h. As shown in Figure 3, compared with the control group, miR-155 expression levels in the ox-LDL group were significantly increased (*p* < 0.05). The data demonstrated that ox-LDL induced the upregulation of miR-155 in HUVECs.

### 3.3. miR-155 Regulates Autophagy Induced by ox-LDL in HUVECs

#### 3.3.1. Effect of miR-155 on the Ultrastructure Changes of Cells

**Figure 4:**
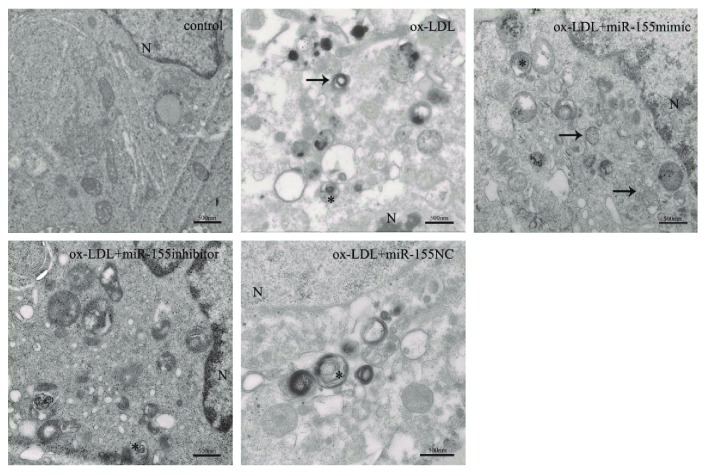
miR-155 promotes autophagosome and autolysosome accumulation. After transfection with miR-155 mimics, miR-155 inhibitors, and miR-155 NC, cells were exposed to 100 *μ*g/ml ox-LDL for 6 h and collected for TEM analysis. Autophagosomes were indicated by arrows. Autolysosomes were indicated by asterisks. N: nucleus. Scale bar = 500 nm.

From the TEM results (Figure 4()), the control group displayed no autophagosomes and autolysosomes. Compared with the control group, single treatment with ox-LDL exhibited apparent autophagosomes and autolysosomes. When cells were transfected with miR-155 mimic, the number of autophagosomes and autolysosomes further increased, whereas transfection with the miR-155 inhibitor reduced the number of autophagosomes and autolysosomes.

#### 3.3.2. Effect of miR-155 on LC3 and p62 Expression

**Figure 5:**
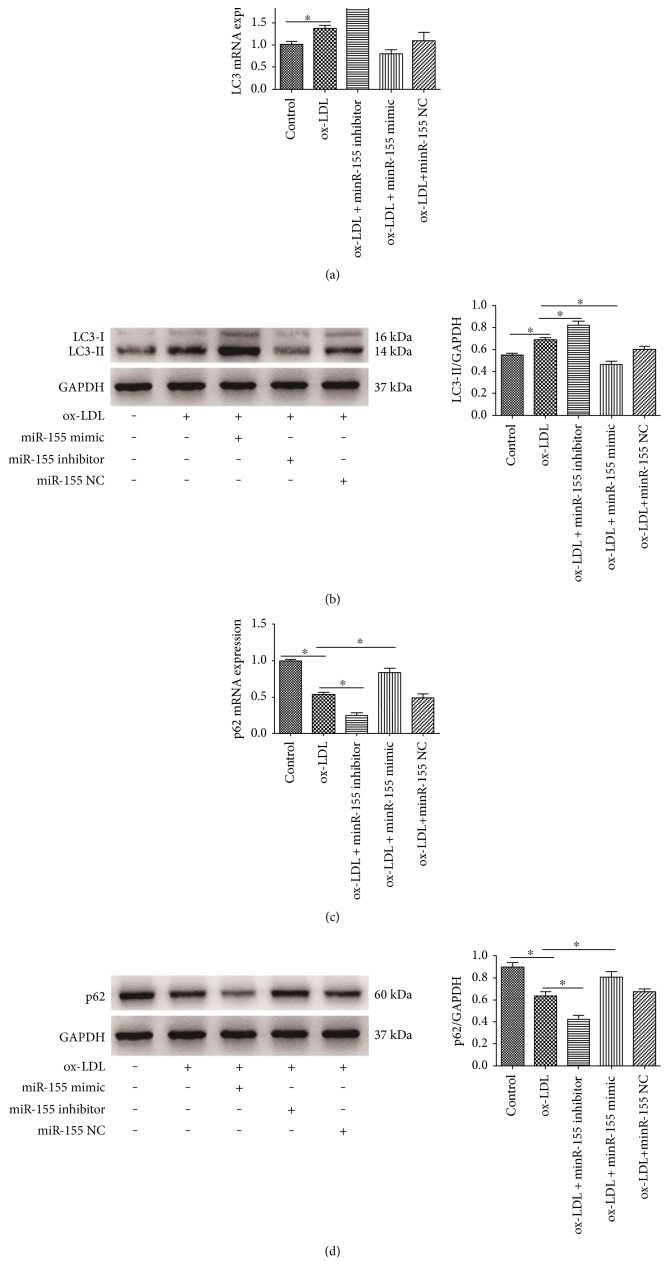
miR-155 regulates LC3 and p62 expression. HUVECs were transfected with miR-155 mimic, miR-155 inhibitor, and mir-155NC and exposed to ox-LDL (100 *μ*g/ml) for 6 h. Cells were harvested for qRT-PCR to detect the expression of mRNA and for Western blot to quantify the protein expression level. (a, c) qRT-PCR analysis of LC3 and p62. Results are the means ± SD of three separate experiments, ^∗^*p* < 0.05. (b, d) Western blot analysis of LC3 and p62. Left panels present representative blots of LC3-II and p62; right bar graphs showed statistics of optical density measurements. Results are the means ± SD of three separate experiments, ^∗^*p* < 0.05.

qRT-PCR results showed that the upregulation of miR-155 enhanced LC3 mRNA levels, whereas the downregulation of miR-155 reduced LC3 mRNA levels (Figure 5(a)). Western blot results indicated that the miR-155 mimic improved the LC3-II/GAPDH ratio, but the miR-155 inhibitor decreased the ratio of LC3-II/GAPDH (Figure 5(b)). We determined p62 mRNA expression levels, and the results showed that p62 mRNA levels were reduced by the miR-155 mimic but improved by the miR-155 inhibitor (Figure 5(c)). In addition, miR-155 enhancement downregulates the ratio of p62/GAPDH, whereas miR-155 suppression upregulated the p62/GAPDH ratio (Figure 5(d)). Taken together, the data above indicated that the overexpression of miR-155 increased the autophagy level; however, the inhibition of miR-155 suppressed the level of autophagy.

## 4. Discussion

As a risk factor of AS, ox-LDL can promote endothelial cell injury. Previous studies have indicated that ox-LDL can result in autophagy in endothelial cells. Nowicki et al. [20] reported that many autophagosomes with double membranes were observed in the EA.hy926 endothelial cells treated with ox-LDL for 6 h. After 12 h of ox-LDL stimulation, cells revealed many autophagic vacuoles with remnants of organelle inclusions. However, there were numerous large vacuoles after 24 h, but seldom with remnants of organelle inclusions. In addition, Zhang et al. [21] found that ox-LDL upregulated the expression of LC3-II and beclin1 in umbilical vein endothelial cells, and the upregulation of proteins reached two peaks at 0.5 and 6 h and then declines at 48 h. Similarly, our study found that ox-LDL (100 *μ*g/ml) increased the number of autophagosomes and the level of the protein LC3-II yet decreased the level of protein p62, which demonstrates that ox-LDL induces autophagy in HUVECs. The present study also confirmed that ox-LDL induced the upregulation of miR-155 in cells. At 6 h and 12 h after ox-LDL (100 *μ*g/ml) treatment, increased miR-155 expression levels were detected. This result suggested that miR-155 might have a regulatory effect on ox-LDL-induced autophagy. Therefore, to further explore the role of miR-155 in autophagy, we intervened the miR-155 level through transfecting the miR-155 mimics and inhibitors into cells. The results showed that the miR-155 mimic increased the number of autophagic bodies and the ratio of LC3-II/GAPDH yet decreased the expression of p62. In contrast, the miR-155 inhibitor downregulated the number of autophagosomes and the expression level of LC3-II but improved p62 expression levels. As a result, the collective data demonstrated that miR-155-enhanced autophagy induced by ox-LDL.

In previous studies, the correlation between miR-155 and autophagy has been studied in other cell types. Chen et al. [16] reported that the overexpression of miR-155 induces the activation of autophagy, which promotes tumor cell survival and chemoresistance. Wang et al. [17] reported that miR-155 promotes autophagy in macrophages by targeting Rheb (a negative regulator of autophagy), conferring protection against infection with intracellular mycobacteria. In addition, Wu et al. [22] demonstrated that the overexpression of miR-155 by transfection with miR-155 mimics significantly decreased the survival of intracellular *Helicobacter pylori* in human gastric epithelial cells, and this process was mediated by the induction of autophagy. However, the relationship between miR-155 and autophagy in endothelial cells has not been reported. Our present results demonstrate that miR-155 promotes ox-LDL-induced autophagy in HUVECs.

Moreover, miR-155 plays a crucial role in AS development, which involves multiple mechanisms. Huang et al. [23] demonstrated that miR-155 serves as a negative feedback regulator in ox-LDL-stimulated THP-1 macrophage inflammatory responses and lipid uptake, which could affect the development of AS. Zhu et al. [24] suggested that miR-155 attenuates apoptosis of ox-LDL-mediated RAW264.7 macrophages by targeting FAS-associated death domain protein (FADD), thus inhibiting the formation of AS plaques. Endothelial cells, as the biological basis of angiogenesis, play a crucial role in the pathogenesis of AS [25]. A growing body of evidence has shown that promoting proper autophagy in endothelial cells may be an effective way to improve AS [26, 27]. Our study found that miR-155 has a promoting effect on ox-LDL-induced autophagy in HUVECs. So, we speculated that miR-155 might have an effect on the process of AS by regulating autophagy in endothelial cells.

In conclusion, the present study indicated that ox-LDL induced autophagy and upregulated miR-155 expression in HUVECs. miR-155 has a promoting effect on ox-LDL-induced autophagy. These findings may be able to provide new ideas for studying the possible role of miR-155 on regulating the process of AS. However, further studies, including in vivo experiments, are needed to confirm this role and the mechanism.
